# Polyaniline Composites Containing Eco-Friendly Biomass Carbon from Agricultural-Waste Coconut Husk for Enhancing Gas Sensor Performance in Hydrogen Sulfide Detection

**DOI:** 10.3390/polym15234554

**Published:** 2023-11-28

**Authors:** Kun-Hao Luo, Minsi Yan, Yu-Han Hung, Jia-Yu Kuang, Hsing-Chih Chang, Ying-Jang Lai, Jui-Ming Yeh

**Affiliations:** 1 Department of Chemistry, Chung Yuan Christian University, Chung Li District‚ Taoyuan City 32023, Taiwan; 2 Department of Food Science, National Quemoy University, Jinning Township, Kinmen County 89250, Taiwan

**Keywords:** polyaniline, biomass activated carbon, H_2_S gas sensor

## Abstract

Hydrogen sulfide, a colorless, flammable gas with a distinct rotten egg odor, poses severe health risks in industrial settings. Sensing hydrogen sulfide is crucial for safeguarding worker safety and preventing potential accidents. This study investigated the gas-sensing performance of an electroactive polymer (i.e., polyaniline, PANI) and its composites with active carbon (AC) (i.e., PANI-AC1 and PANI-AC3) toward H_2_S at room temperature. PANI-AC composites-coated IDE gas sensors were fabricated and their capability of detecting H_2_S at concentrations ranging from 1 ppm to 30 ppm was tested. The superior gas-sensing performance of the PANI-AC composites can be attributed to the increased surface area of the materials, which provided increased active sites for doping processes and enhanced the sensing capability of the composites. Specifically, the incorporation of AC in the PANI matrix resulted in a substantial improvement in the doping process, which led to stronger gas-sensing responses with higher repeatability and higher stability toward H_2_S compared to the neat PANI-coated IDE sensor. Furthermore, the as-prepared IDE gas sensor exhibited the best sensing response toward H_2_S at 60% RH. The use of agricultural-waste coconut husk for the synthesis of these high-performance gas-sensing materials promotes sustainable and eco-friendly practices while improving the detection and monitoring of H_2_S gas in industrial settings.

## 1. Introduction

Because of insufficient internal air circulation, numerous accidents occur in the oil storage, chemical reaction, and solvent distillation units of modern chemical industries during annual maintenance. Hydrogen sulfide (H_2_S), a highly hazardous gas used and generated in this industry, poses a significant risk [1,2]. This colorless, corrosive, explosive, and flammable acid gas is highly soluble in water and produces hydrosulfuric acid [3]. The severity of the health effects of H_2_S varies depending on its concentration. At low concentrations (~1.5 ppm), H_2_S has a rotten egg odor and can cause chronic symptoms, such as memory loss [4], facial paralysis [5], and nerve tissue damage [6], but is not life-threatening. By contrast, at high concentrations, H_2_S is extremely dangerous. At concentrations >140 ppm, H_2_S can paralyze the olfactory system; exposure to H_2_S concentrations ≥700 ppm can be fatal. Short-term exposure to high levels of H_2_S can also affect various organs [7]. Therefore, H_2_S gas sensors with high sensitivity and high selectivity are crucial.

Various semiconducting materials, spanning both inorganic and organic compounds, have been investigated for the advancement of H_2_S sensors. Notably, inorganic materials like zinc oxide [8], copper oxide [9], tin oxide [10], indium oxide [11], and cobalt ferrite [12] showcase commendable H_2_S-sensing properties in an operating temperature ranging from 75 °C to 400 °C. However, this temperature range means that inorganic materials are not suitable for ambient working conditions where human protection from H_2_S gas poisoning is essential [13]. Recent research trends have pivoted towards organic sensors, especially those employing conductive polymers, seeking to mitigate the temperature dependency observed in inorganic analogs [14,15].

Among the diverse conductive polymers explored, polyaniline (PANI) has emerged prominently, lauded for its outstanding environmental stability, reversible redox capacity, and reversible doping capability, setting it apart in the realm of conductive polymers. Significantly, Monkman et al. [16] ushered in a pivotal era in 1995 by introducing the concept of coating a thin PANI film onto an interdigitated electrode (IDE) on a quartz substrate for H_2_S sensing through a doping mechanism, anchoring the historical significance of PANI in gas sensor development. Furthermore, the utilization of HCl-doped PANI-related materials for ammonia (NH_3_) sensing through a dedoping mechanism extended the narrative of conductive polymer applications in gas sensing. These contributions add a nuanced layer to the diverse landscape of conductive polymer applications, showcasing the versatility and potential of PANI in H_2_S and NH_3_ gas-sensing scenarios. This unique amalgamation of properties positions PANI as a compelling candidate for gas-sensing applications [17,18,19].

As a main source of carbon derivation, sustainable natural materials such as clay minerals [20] and soil minerals [21] have gained global prominence as primary sources for carbon derivation, sparking a surge in applications for renewable energy. Biomass, encompassing organic matter derived from various sources, including agricultural crops [22], forest residues [23], animal wastes [24], and industrial by-products [25], serves as a crucial renewable energy source. Despite biomass being a primary resource distinct from the aforementioned minerals, its utilization has witnessed a recent upswing due to its capacity to mitigate greenhouse gas emissions and foster sustainable development [26,27]. When biomass is pyrolyzed at low temperatures (i.e., 200 °C–300 °C) in the presence of air or nitrogen gas, biochar, with a low porosity and a small specific surface area, is produced [28]. Biomass can be further activated and transformed into nanoporous carbon materials with a large surface area and well-developed porosity, which can be leveraged to enhance the performance of gas sensors [29]. This activation can be achieved through physical or chemical activation methods [30,31]. The physical activation of biochar at high temperatures (i.e., 800 °C–1100 °C) using steam or CO_2_ is a simple and cost-effective method to produce nanoporous activated carbons (ACs) with specific surface areas in the range of 500–1000 m^2^/g. Chemical activation can further enhance the specific surface area to >1000 m^2^/g [32]. The chemical activation process involves impregnating the biomass or biochar with an activating agent, followed by carbonization in an inert atmosphere of nitrogen or argon gas at temperatures ranging from 400 °C to 1000 °C [33]. Activating agents, usually alkali and alkaline earth metal-containing substances and some acids, such as KOH, K_2_CO_3_, NaOH, Na_2_CO_3_, AlCl_3_, ZnCl_2_, MgCl_2_, H_3_PO_4_, and H_2_SO_4_, can be used to enhance porosity through the depolymerization and dehydration of biochar [34]. ZnCl_2_ is a commonly used activating agent that contributes to the creation of a porous structure by acting as a template. ZnCl_2_ dehydrates and accelerates the decomposition of carbonaceous materials during the carbonization process, restricts the formation of tar, and intercalates in the carbon matrix to create void spaces. The incorporation of biomass-derived materials, such as carbon nanotubes [35] and graphene oxide [36], in gas-sensing materials has been shown to improve the sensitivity, selectivity, and stability of gas sensors, including those sensors used for detecting harmful gases such as H_2_S [37,38].

Leveraging the favorable properties of PANI and AC, the PANI-AC hybrid composites were prepared by physical mixing of chemically synthesized PANI with biomass carbon. While recent in situ polymerization methods have been applied to synthesize PANI-based carbon material composites [39,40,41], their synergy faces inherent challenges. In such cases, molecular chains of the polymer may hinder filler interconnection, counteracting intended synergies [42]. The nature of in situ polymerization also restricts bulk production. Opting for an alternative, simpler, and cost-effective method like physical mixing not only overcomes these limitations but also enhances the formation of hybrid structures. This approach holds promise for tailoring composites with superior electrical and chemical properties, expanding their applications in gas sensors, particularly for industrial production [43].

In this study, PANI has been incorporated with AC, a natural biomass derived from coconut husks, to fabricate H_2_S sensors exhibiting a higher response value, great repeatability, and high stability. AC increases the specific surface area of the material, improving its redox capability and substantially enhancing the gas-sensing performance of the resulting sensor. The use of biomass in the sensor used for sensing H_2_S demonstrates the enormous potential of biomass in improving the performance of existing sensors and developing new sensing materials. The utilization of agricultural waste in the development of sensing materials can also contribute to a highly sustainable and environmentally friendly approach toward agricultural-waste management.

## 2. Experimental Section

### 2.1. Chemicals

Aniline (99.5%; Acros Organics, Geel, UK) was distilled at 70 °C under 0.1 atm pressure before use. N-methyl-2-pyrrolidone (NMP; 99%; Duksan, Seoul, Republic of Korea), ammonium persulfate (APS; 97.0%; J.T. Baker, Radnor, PA, USA), methanol (99%; Sigma-Aldrich, Burlington, VT, USA), ZnCl_2_ (98%; Sigma-Aldrich, Burlington, VT, USA), isopropanol (99.5%; Sigma-Aldrich, Burlington, VT, USA), hydrochloric acid (HCl; 37%; Riedel-de Haen, Seelze, Germany), and ammonium hydroxide (NH_4_OH; 28%; Riedel-de Haen, Seelze, Germany) were used as received without further purification. All gases used in this study, including H_2_S, SO_2_, CO_2_, NO_2_, NH_3_, CO, and air (50 ppm; Chian Hong Gas Co., Ltd., Hong Kong, China), were used as received. An Interdigitated Electrode device (IDE, thickness of 0.375 mm, 12 pairs of electrodes, spacing of 0.3 mm, and length × width of 20 × 20 mm) was fabricated using indium tin oxide (ITO), purchased from Ruilong Optoelectronics in Miaoli, Taiwan, for the gas-sensing study.

### 2.2. Instruments

A laser engraver (Flux Beambox, Taipei, Taiwan) was employed to carve grooves into the ITO glass to form an ITO-based IDE that resulted in nonconductive open circuits on both sides of the glass. Raman scattering spectra were acquired using a Raman spectrometer (Horiba Jobin Yvon, Traix 320, Palaiseau, France) with a laser source using an excitation wavelength of 640 nm. A Fourier-transform infrared (FTIR) spectrometer (JASCO, FT/IR-4200, Tokyo, Japan) was employed to acquire the FTIR spectra of calcined carbon (CC) and AC at room temperature and for wavelengths ranging from 4000 to 400 cm^−1^. Moreover, the spectra of PANI and PANI-AC composites were obtained using the attenuated total reflectance mode of FTIR (ATR-FTIR) at room temperature and for wavelengths ranging from 4000 to 600 cm^−1^. A PFY400 oven (Dengyng Instruments Co., Ltd., Kaohsiung, Taiwan) was utilized for the preparation of CC and AC. Cyclic voltammetry (CV) measurements were conducted using the AutoLab PGSTAT204 (Ω Metrohm Autolab, KM Utrecht, The Netherlands) electrochemical workstation. A four-point probe connected to a voltmeter system with a constant current source (Keithley 2400, Tektronix, Beaverton, OR, USA) was used to evaluate the conductivity of PANI-AC composites and determine the conductivity of the undoped, doped, and de-doped forms of PANI and PANI-AC composites. The morphology and composition of the composites were determined via scanning electron microscopy (SEM; JEOL JSM-7600F, Tokyo, Japan). The N_2_ adsorption–desorption isotherm at 77K (Brunauer–Emmett–Teller [BET] analysis; Micromeritics ASAP-2010, Norcross, GA, USA) was used to calculate the surface area and pore diameter of samples. Conductivity measurements of the sensors were performed using a four-point probe (LRS4-TK1, KeithLink Technology, Taipei, Taiwan). The fabrication of IDEs was accomplished using a laser engraver (Flux Beambox, Taipei, Taiwan). The H_2_S-sensing capabilities of all PANI-composite-coated IDE sensors were evaluated using a custom-made sensing device in our laboratory. To measure the sensor response, an electrometer (Keithley 2450 Source Meter, Keithlink Technology, Taipei, Taiwan) was employed. The carrier gas (air) was combined with the target gas from a cylinder and maintained at a fixed concentration of 50 ppm. This gas mixture was subsequently directed through a programmable mass-flow controller (MFC; F-201CB Mass-flow Controller, Bronkhorst, Ruurlo, The Netherlands). The target and carrier gases were simultaneously introduced into the mixing chamber, enabling the generation of different analyte concentrations.

### 2.3. Synthesis of PANI

**Scheme 1:**
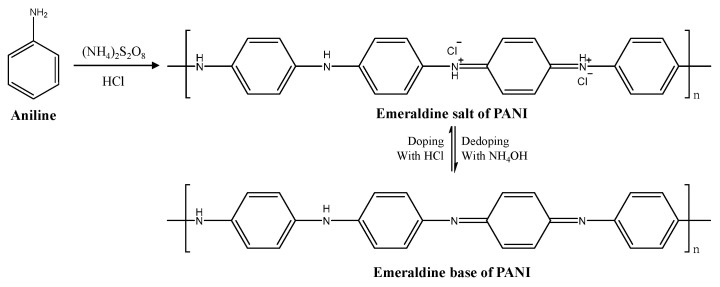
Synthesis of PANI and its reversible doping behavior.

PANI was synthesized via oxidation coupling polymerization [44], as shown in Scheme 1. First, 0.1 mol of aniline monomer was dispersed perfectly under magnetic stirring in 400 mL of 1.0 M HCl. Then, 0.025 mol of APS was added to 20 mL of 1.0 M HCl and the resultant solution was stirred in an ice bath for 6 h. This resultant solution was added quickly to the precooled aniline solution, and the resulting mixture was stirred at 10 °C for 24 h to promote polymerization. The precipitate obtained through suction filtration underwent washing with 400 mL of 0.1 M NH_4_OH, with magnetic stirring maintained for 4 h at room temperature. Subsequently, the mixture was filtered and vacuum-dried for 2 days at 40 °C to yield the final PANI products.

### 2.4. Preparation of CC and AC Using Agricultural-Waste Coconut Husk

**Scheme 2:**
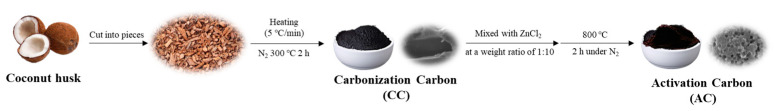
Flow chart for the preparation of CC and AC.

Scheme 2 illustrates the preparation process of CC and AC. Natural biomass waste was initially washed three times with distilled water and subsequently dried at 60 °C overnight in an oven. Then, the dried biomass waste was cut into small pieces and subjected to precarbonization by heating at 300 °C with a heating rate of 5 °C min^−1^ for 2 h under a N_2_ atmosphere. The resulting precarbonized biochar was ground and further washed with methanol, ethanol, and distilled water before drying at 80 °C. In the activation step, the precarbonized biochar was mixed with ZnCl_2_ at a weight ratio of 1:10 and carbonized at 800 °C for 2 h under a N_2_ flow in a tube furnace. The temperature ramp during carbonization was set to 5 °C min^−1^. Finally, the synthesized AC was washed with a mixture of water and isopropanol (1:1) and dried under vacuum at 80 °C for 6 h [45].

### 2.5. Preparation of PANI-AC Composites

A straightforward physical mixing method was employed for the synthesis of PANI composites incorporating coconut-husk-derived AC. Initially, a 1 wt% PANI solution was created by dissolving 1 g of PANI in 9 g of NMP under magnetic stirring at room temperature. Subsequently, different weights of AC (0.01 g, constituting 1% of PANI, and 0.03 g, constituting 3% of PANI) were introduced into the prepared solution, followed by overnight stirring. The resulting composites, labeled as PANI-AC1 and PANI-AC3, were obtained through suction filtration and subjected to a 2-day vacuum-drying process at 40 °C.

### 2.6. Preparation of PANI-AC Composites-Coated IDE Sensors

**Scheme 3:**
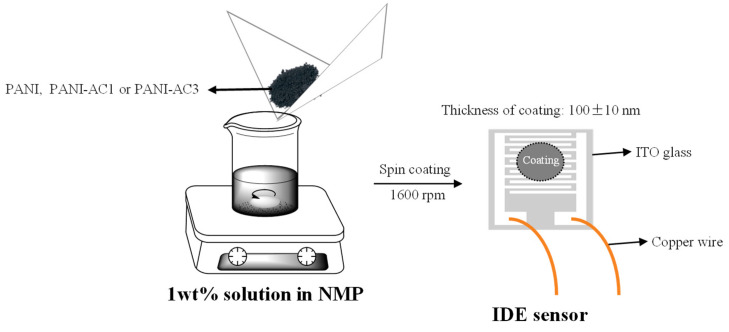
Preparation of PANI, PANI-AC1, and PANI-AC3-coated IDE sensors for measuring gas-sensing properties.

The spin-coating technique was used to prepare IDE sensors coated with PANI, PANI-AC1, and PANI-AC3. First, 1 wt% solutions of the samples of PANI, PANI-AC1, and PANI-AC3 were prepared using NMP under stirring at room temperature, as shown in Scheme 3. Thereafter, thin films were prepared by spin coating 200 μL of the corresponding solutions on ITO at a spin rate of 1600 rpm followed by drying overnight at room temperature. An advantage of spin coating is that it produces extremely fine, thin, and uniform coatings. Using the spin-coating method, the desired film thickness (100 ± 10 nm) can be achieved.

### 2.7. Gas-Sensing Experiment

**Scheme 4:**
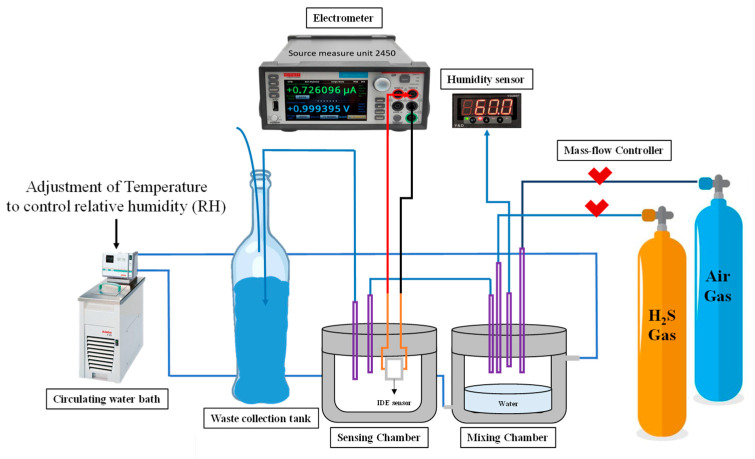
Schematic diagram of the gas-sensing apparatus.

In the gas-sensing experiment, PANI-coated, PANI-AC1-coated, and PANI-AC3-coated IDE sensors were exposed to various concentrations of the target gas (ranging from 1 ppm to 30 ppm) at room temperature. The target gases are H_2_S and five other common toxic gases, namely SO_2_, CO_2_, CO, NH_3_, and NO_2_. The IDE sensor was fixed in a gas chamber and connected to an electrometer to measure the responses, as depicted in Scheme 4. All measurements were conducted at a stable state after reaching room temperature (i.e., 25 ± 0.5 °C). The modulation of relative humidity (RH) to levels of 40%, 60%, and 80% was accomplished by regulating the temperature of the water in the mixing chamber. This control was facilitated through a circulating water bath connected to the mixing chamber, with alterations tracked by a humidity sensor positioned within the mixing chamber. The carrier gas (air) was mixed with the target gas in the mixing chamber, and the gas concentration was controlled by the programmable MFC to investigate the dependency of PANI-composite-coated IDE responses on gas concentration. The gas chamber was set to a total-gas-flow value of 1000 sccm. Each measurement was performed by flushing the measurement chamber with H_2_S gas for 150 s, followed by cleaning the sensor with air until the baseline was reached. The responses of PANI-composite-coated IDE sensors were evaluated as the normalized resistance (R_a_/R_g_), where R_a_ and R_g_ indicate the resistance under air and the given analyte, respectively. After the gas-sensing experiment, the gas was collected in a waste collection tank filled with water to dilute its toxicity.

## 3. Results and Discussion

### 3.1. Material Characterization

#### 3.1.1. Raman Analyses of CC and AC

**Figure 1:**
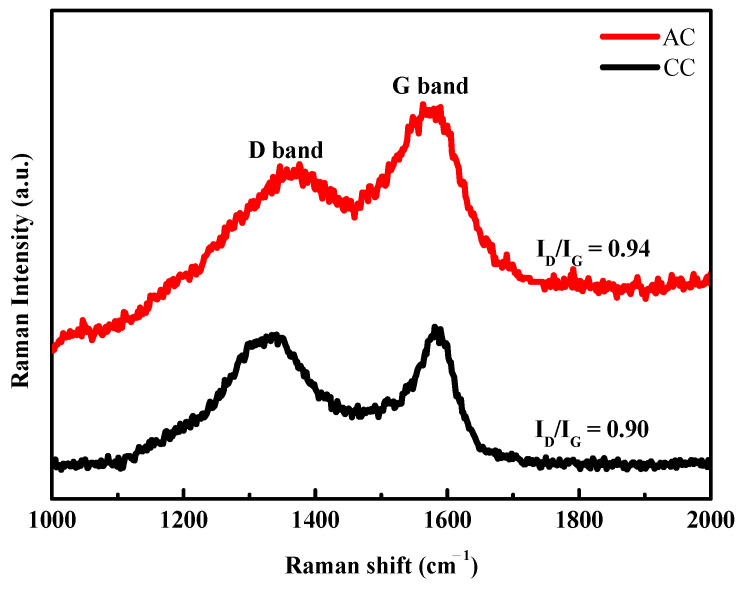
Raman spectra of CC and AC.

Figure 1 shows the Raman spectra of CC and AC. The D band was located at ~1330 cm^−1^, indicating the presence of disordered structural defects due to carbonization [45]. The G band appeared at 1580 cm^−1^, which was attributed to the ordered nature of any carbon atoms with a hexagonal two-dimensional structure because of C=C bond stretching. The D band in the CC spectrum corresponded to the double resonance effect of disordered carbon structures, whereas the G band corresponded to the in-plane vibration of ordered graphite. The relative intensity integration ratio of the D and G bands (*I*_D_/*I*_G_) with respect to AC indicated an increase in carbon defects owing to the structural damage caused to the material after activation [46].

#### 3.1.2. FTIR Analyses of CC, AC, PANI, and PANI-AC Composites

**Figure 2:**
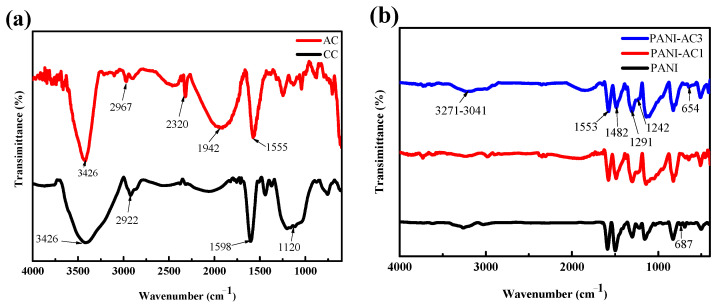
(**a**) FTIR spectra of CC, AC; (**b**) ATR-FTIR spectra of PANI, PANI-AC1, and PANI-AC3.

A broad absorption band ranging from 3000 to 3700 cm^−1^, with a peak appearing between 3420 and 3440 cm^−1^, is evident in the FTIR spectra of CC and AC (Figure 2a). This band was attributable to the O–H stretching of the phenolic groups [47]. The peaks observed in the range of 2922–2967 cm^−1^ were attributed to the stretching vibrations of the C–H bond [48]. The peaks at 2320 and 1550 cm^−1^ correspond to the stretching vibration of the C=O bond in carboxylic acid, whereas that at 1120 cm^−1^ was attributed to the stretching vibration of the C–O bond [49]. After activation, the peaks at 2967 and 1555 cm^−1^ can be attributed to the C–H and C=C stretching frequencies, respectively, indicating the common graphite-like extended conjugation of AC [50]. The peaks detected for the PANI-AC1 and PANI-AC3 composites were also visible in the ATR-FTIR spectra of PANI, as shown in Figure 2b. In the spectrum of the PANI-AC composite, the peak observed between 3271 and 3041 cm^−1^ represented the stretching vibration of the N–H bond. The characteristic peak observed at 1553 cm^−1^ corresponded to the quinoid ring, whereas the peak at 1482 cm^−1^ corresponded to the benzenoid group. The incorporation of AC resulted in a peak similar to that of pure PANI, with a slight difference in peak intensity. PANI-AC composites exhibited an inverse intensity ratio of the bands at 1553/1482 cm^−1^ compared to pure PANI, indicating a higher abundance of quinoid units in PANI-AC composites. This suggested longer conjugation lengths in the polymeric chains of PANI-AC composites. The conjugated structure of PANI tended to strongly interact with the AC surface, particularly with the quinoid ring. Such aromatic structures were well-known for their ability to interact with the graphitic surface through π–π stacking [51]. The peak at 1291 cm^−1^ represented the C=C stretching vibration. The peak at 1242 cm^−1^ represented the C–N stretching vibrations of the benzenoid and quinoid rings. The peak at 654 cm^−1^ indicated out-of-plane vibration related to the C–H bending in the phenylene rings shifted to higher regions at 654 cm^−1^ [52].

### 3.2. Pore Structure Characterization via the BET Analyses of CC, AC, PANI, and PANI-AC Composites

**Figure 3:**
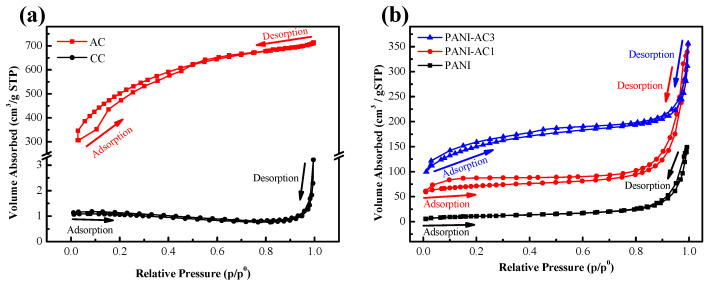
(**a**) N2 (77K) adsorption/desorption isotherms of (**a**) CC and AC; (**b**) PANI, PANI-AC1, and PANI-AC3.

This study employed N_2_ adsorption–desorption analysis at 77 K to investigate the specific surface area, pore structure, and average pore diameter of the samples, as illustrated in Figure 3. Table 1 provides the BET surface area values for CC and AC, which were determined to be 5 and 1718 m^2^/g, respectively. The carbonized coconut husk exhibited a relatively small surface area, a common characteristic in lignocellulosic materials. This phenomenon is attributed to particle aggregation and unchanged porosity, resulting in smooth surfaces [53]. As shown in Figure 3a, the adsorption/desorption curves of CC revealed a Type II isotherm typical for macroporous materials, with an average pore size of 42.7 nm. In contrast, AC exhibited a Type I isotherm, indicating that the pores were not considerably larger than the diameter of the adsorbate, and the pore size was measured to be 2.6 nm. These findings underscored the significance of the activation process for CC. During pyrolysis, nanoporous carbon materials are formed, and the gaseous materials released in the subsequent stage act as pore generators, ultimately leading to the development of mesopores and an increase in the surface area of CC [54]. Figure 3b illustrates the N_2_ adsorption–desorption curves of the PANI and PANI-AC composites, displaying a Type IV isotherm with an H3 hysteresis loop. This characteristic signifies the porous structure of PANI and PANI-AC composites [55]. For PANI, PANI-AC1, and PANI-AC3, the results presented in Table 1 show that the BET surface area of the samples increased with the incorporation of AC. Specifically, the BET surface areas of PANI-AC1 and PANI-AC3, measuring 225 and 416 m^2^/g, respectively, were substantially larger than that of pure PANI, measuring 38 m^2^/g. This increase in the specific surface areas of PANI-AC1 and PANI-AC3 can be attributed to the porous structure of AC, which provides additional active sites for interaction with H^+^ molecules dissociated from H_2_S [56].

### 3.3. Morphologies of CC, AC, PANI, and PANI-AC Composites

**Figure 4:**
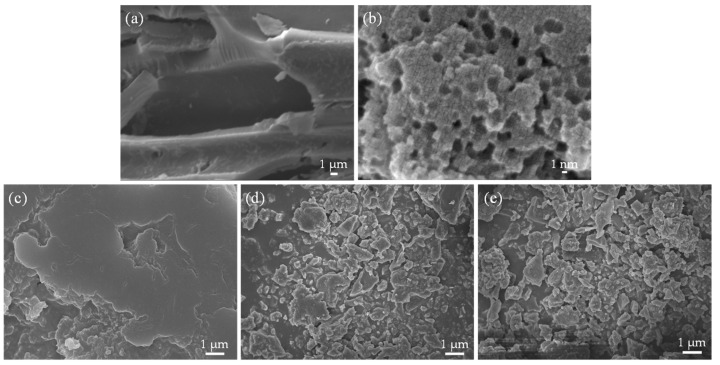
SEM image of the surface structure of the (**a**) CC, with a magnification of 10,000, (**b**) AC, with a magnification of 300,000; SEM image of the surface structure of PANI and PANI composites, with a magnification of 10,000. (**c**) Structure diagram of PANI, (**d**) PANI-AC1, and (**e**) PANI-AC3.

Figure 4a shows the original shape of coconut shell–based carbon powder after carbonization, and stacked microsheet structures were observed. Figure 4b illustrates AC with visible pores that are critical for increasing the specific surface area. These pores were nonexistent in CC without the activating agent, ZnCl_2_, because oxidation did not occur on the carbon surface in CC. This study investigated the effect of AC on the morphology of PANI composites. The SEM image of PANI (Figure 4c) shows a smooth surface morphology without any distinctive features. However, in Figure 4d,e, a substantial modification in the morphology of PANI is observed owing to the incorporation of AC. The absence of agglomeration in the SEM images of PANI-AC1 and PANI-AC3 is consistent with the BET analyses that demonstrate that the addition of AC can enhance the formation of composites having improved properties, such as a large specific surface area. These results indicate that the incorporation of AC can induce desirable changes in the morphology of PANI, which may have implications related to the potential applications of AC in gas sensing.

### 3.4. Electrochemical Redox Properties of PANI-AC Composites

**Figure 5:**
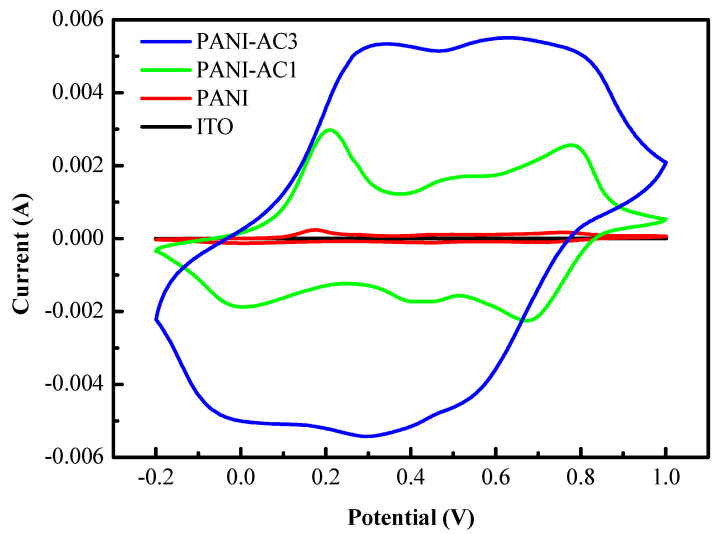
Cyclic voltammogram showing the redox capabilities of PANI, PANI-AC1, and PANI-AC3.

CV performance was investigated to evaluate the redox properties of each component in the potential range of −0.2 V to 1.0 V in 1.0 M H_2_SO_4_ at various scan rates, and the scan rate of 5 mV/s was selected for comparison in Figure 5 [57]. Three pairs of redox peaks were observed for PANI, PANI-AC1, and PANI-AC3, where all oxidation peaks were accompanied by a reduction peak, which was associated with the main chain of PANI that can exist in four oxidation states, i.e., Leucoemeraldine, Emeraldine I, Emeraldine II, and Pernigraniline [58]. The oxidation and reduction peak pairs appeared at 0.17/−1.25 V, 0.45/0.44 V, and 0.75/0.72 V for PANI. For PANI-AC1, the oxidation and reduction peak pairs appeared at 0.21/0.01 V, 0.51/0.41 V, and 0.78/0.68 V. For PANI-AC3, the oxidation and reduction peak pairs appeared at 0.31/0.54 V, 0.62/0.30 V, and 0.81/−0.05 V. Importantly, the incorporation of AC in the PANI matrix caused a shift in the oxidation peaks toward higher currents, indicating more effective electron inclusion and exclusion during the redox processes in the PANI-AC composites than in the neat electroactive polymer. This improvement was attributed to the enhancement of the surface area by AC that led to increased active sites that benefited the redox processes [59].

### 3.5. Electrochemical Doping Properties of PANI-AC Composites

The impact of increased surface area in PANI-AC composites on their doping capacity was assessed using a four-point probe technique with hydrosulfuric acid as the dopant. The conductivities of undoped, doped, dedoped, and redoped PANI-AC composites were determined (Table 2). These results highlighted the reversible doping–dedoping capability of PANI, with similar observations in PANI-AC composites. Notably, the conductivities of the doped and redoped forms of PANI-AC composites, especially PANI-AC3, were significantly higher, approximately 10 times more than those of the corresponding PANI forms. This difference is attributed to the incorporation of AC, which provides a larger surface area than bare PANI, offering numerous active sites for dopants. The increased conductivity of PANI-AC composites during doping suggests a superior gas-sensing performance compared to PANI alone.

### 3.6. Gas-Sensing Performance

#### 3.6.1. Sensitivity and Effect of Humidity

**Figure 6:**
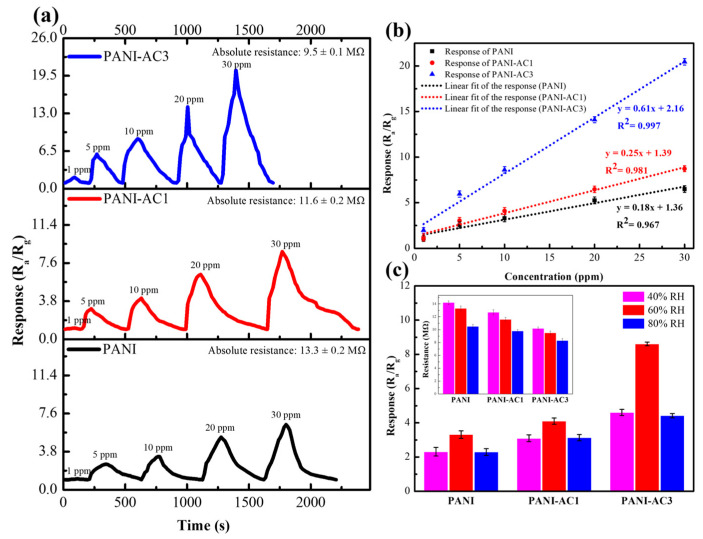
(**a**) Sensitivity of the IDE sensor device with the PANI, PANI-AC1, and PANI-AC3 coating at various concentrations of H_2_S ranging from 1 ppm to 30 ppm, respectively. (**b**) Linear calibration curves of the aforementioned IDE sensors exposed to 1–30 ppm H_2_S concentration at +0.1 V. (**c**) Comparison response of the aforementioned IDE sensors exposed to 10 ppm H_2_S with 40%, 60%, and 80% RH. Inset: comparison response of the aforementioned IDE sensors exposed to 10 ppm H_2_S with 40%, 60%, and 80% RH.

The IDE sensor responses were examined at different H_2_S concentrations (i.e., 1, 5, 10, 20, and 30 ppm), as presented in Figure 6a. The absolute resistance of PANI-coated, PANI-AC1-coated, and PANI-AC3-coated IDEs was 13.3 ± 0.2, 11.6 ± 0.2, and 9.5 ± 0.1 MΩ, respectively. The response signals of PANI-coated, PANI-AC1-coated, and PANI-AC3-coated IDEs increased gradually with the increase in H_2_S concentration. The response signals exhibited a gradual increase in PANI-coated, PANI-AC1-coated, and PANI-AC3-coated IDEs with the elevation of H_2_S concentration. As H_2_S gas was introduced, the PANI and PANI-AC composites underwent doping with hydrosulfuric acid as the dopant, inducing a change in conductivity and a subsequent sharp decrease in resistance as a response. Notably, variations in H_2_S concentration directly influenced the response, with higher concentrations of dopant correlating to more pronounced responses. Subsequent replacement with air facilitated the removal of the dopant by the air, causing the response to revert to the baseline state. This analysis underscores the close relationship between acidic gas doping, gas concentration, and the resulting gas-sensing characteristics in PANI and PANI-AC composites. For the PANI sensor, the response values for detecting 1, 5, 10, 20, and 30 ppm H_2_S gas were 1.09, 2.55, 3.31, 5.25, and 6.50, respectively. The PANI-AC1 sensor responses were even higher, reaching 1.12, 3.04, 4.10, 6.47, and 8.73 for the same concentrations. Meanwhile, the PANI-AC3 sensor showed the highest response values at 1.80, 5.96, 8.61, 14.09, and 20.44 for the same concentrations. The PANI-AC3-coated IDE sensor exhibited a stronger response than the PANI-coated IDE sensor. Notably, the difference in sensor response could be attributed to the introduction of AC, which improved the doping properties via the availability of a larger number of active sites in the sensing layer to provide doping reaction sites for H^+^ dissociated from H_2_S.

Figure 6b displays the linear calibration curves of the PANI-coated, PANI-AC1-coated, and PANI-AC3-coated IDE sensors exposed to different concentrations of H_2_S. The linear fit equations were y = 0.18x + 1.36, y = 0.25x + 1.39, and y = 0.61x + 2.16 for the PANI-coated, PANI-AC1-coated, and PANI-AC3-coated IDE sensors, respectively. The correlation coefficients for the fitted data (R^2^) were 0.967, 0.981, and 0.997 for the PANI-coated, PANI-AC1-coated, and PANI-AC3-coated IDE sensors, respectively. Notably, a highly linear response was observed in the tested H_2_S range, where the R^2^ of the PANI-AC3-coated IDE sensor was higher than that of the PANI-coated IDE sensor, indicating the reliable calculation and superiority of PANI-AC3 in gas sensing.

To investigate the effect of humidity on the sensing of toxic gases, the responses of the PANI-coated, PANI-AC1-coated, and PANI-AC3-coated IDE sensors at a concentration of 10 ppm were compared, as depicted in Figure 6c. The responses of the PANI-coated, PANI-AC1-coated, and PANI-AC3-coated IDE sensors at 40% and 80% RH were observed to be lower than those at 60% RH. The decrease in sensor response at 40% RH was attributed to the insufficient adsorption of water molecules on the material, thereby reducing the number of active protonic acid sites. By contrast, the decrease in sensor response at 80% RH was due to the overabundance of adsorbed water molecules, leading to the occupation of the active sites by water vapor. Both effects resulted in a decrease in sensor response [60]. In the inset of Figure 6c, a decrease in the absolute resistance of the gas sensor is observed with an increase in RH, which can be attributed to the further protonation of PANI through the adsorption of water or the formation of H_3_O^+^ [61].

#### 3.6.2. Repeatability, Selectivity, and Stability

**Figure 7:**
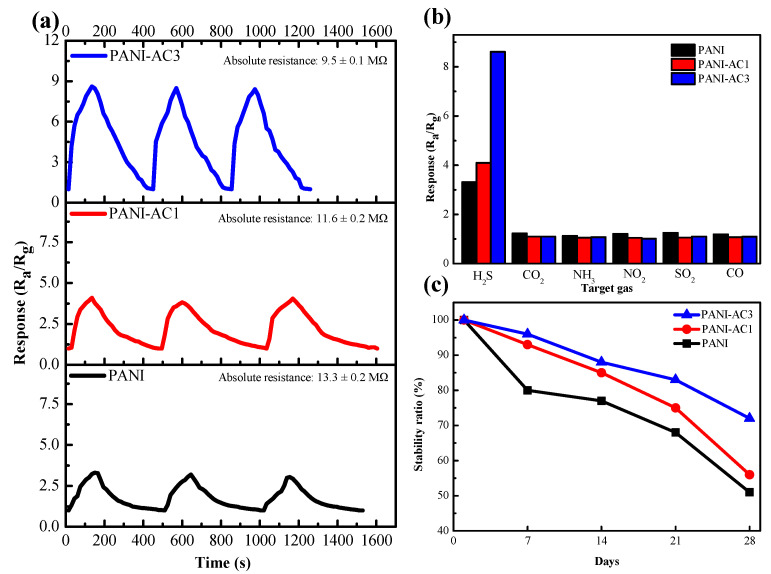
(**a**) Repeatability of the PANI, PANI-AC1, and PANI-AC3-coated IDE sensors at an H_2_S concentration of 10 ppm. (**b**) Selectivity of the aforementioned sensors exposed to six different gases (concentration = 10 ppm). (**c**) Stability of the aforementioned sensors (concentration = 10 ppm).

The repeatability of PANI-coated, PANI-AC1-coated, and PANI-AC3-coated IDE sensors was evaluated by exposing the devices to an H_2_S concentration of 10 ppm for three adsorption–desorption cycles, as shown in Figure 7a. The PANI-AC3-coated IDE sensor exhibited highly repeatable and constant responses throughout each cycle, indicating its excellent repeatability. By contrast, the PANI-coated IDE sensor remained at a consistent lower response throughout the three tests. Furthermore, all sensors fully recovered after multiple adsorption–desorption cycles, indicating the reversibility of H_2_S gas sensing.

To evaluate the selectivity of the as-fabricated sensors, in addition to H_2_S, five other common toxic and hazardous gases, namely CO_2_, NH_3_, NO_2_, SO_2_, and CO, were tested at the same concentration (i.e., 10 ppm). As shown in Figure 7b, the sensors exhibited a higher selectivity toward H_2_S than toward the other gases. Despite the stronger acidity of nitric and sulfuric acids compared to hydrosulfuric acid, NO_2_ and SO_2_ interacted directly with PANI without undergoing dissociation for doping, leading to distinct sensing behaviors [42,43,44]. Notably, the PANI-AC3-coated IDE sensor showed a markedly higher selectivity toward H_2_S than the PANI-coated IDE sensor.

Stability (Figure 7c) was determined by continuously measuring the response values of the PANI-coated, PANI-AC1-coated, and PANI-AC3-coated IDE sensors to 10 ppm H_2_S gas at room temperature for 7, 14, 21, and 28 days. The stability ratio was estimated using Equation (1):

Stability ratio (%) = R_s_/R_s0_ × 100%, (1)

where R_s0_ and R_s_ represent the recorded resistance on the first day and the following days, respectively. The response values of all the sensors decreased with time. After 28 days, the stability ratio of the PANI-coated IDE sensor remained at 51%, whereas those of the PANI-AC1-coated and PANI-AC3-coated IDE sensors were as high as 56% and 72%, respectively. This result indicates the robustness of the sensor, which provides reproducible results within 28 days, and the stability improved with the increasing load of AC.

In Table 3, the sensing properties of H_2_S gas sensors based on a variety of conductive polymers and composites are summarized. Among these materials, the PANI-AC3 composite developed in this study stands out with its notable response to H_2_S gas at room temperature, achieving a response value of 8.6 at a concentration of 10 ppm. This promising result indicates that the PANI-AC3 composite has the potential to be a sensitive and reliable material for H_2_S gas-sensing applications. The use of agricultural-waste-derived AC in the composite also demonstrates the potential for sustainable and eco-friendly approaches to material development.

### 3.7. Gas-Sensing Mechanism

The gas-sensing mechanism primarily hinged on the intrinsic properties of PANI, with the reversible doping process playing a pivotal role [70]. A reversible acid/base doping process is often observed, with the doped form being conductive and the dedoped form being insulative. When exposed to H_2_S in a humid environment, it can react with water molecules and dissociate into H^+^ and HS^−^ ions, where the H^+^ ion has doped PANI, increasing the conductivity of the polymer chain by increasing the number of holes in the chain [16]. When PANI is exposed to a reduced concentration of H_2_S, the following doping reactions can occur [60]:

(2) $$H_{2}S\;\overset{H_{2}O}{\to }H^{+}{+\mathrm{HS}}^{-}$$

(3) $$\mathrm{PANI}+H^{+}\to {\mathrm{PANIH}}^{+}$$

In the PANI-AC composite, the incorporation of AC has been found to markedly improve its sensing performance, particularly toward H_2_S. This improvement was attributed to the heightened specific surface area, creating a surplus of active sites for H^+^ molecules, thereby enhancing the doping capability of the material and subsequently amplifying the sensor’s response to H_2_S. The observed enhancements in response values for the PANI-AC composites underscored the efficacy of this approach [59]. Furthermore, the introduction of AC contributed to heightened selectivity and stability, positioning the sensor as a highly promising candidate for practical gas-sensing applications. This emphasized the dominance of doping capability in the gas-sensing mechanism.

## 4. Conclusions

In this study, a sensitive H_2_S gas sensor was successfully fabricated using an IDE with PANI and PANI-AC composite coatings operating at room temperature (i.e., 25 ± 0.5 °C) and 60% relative humidity. PANI was synthesized via the oxidation coupling polymerization of an aniline monomer, whereas AC was synthesized via the calcination of coconut husk, an agricultural waste, at up to 800 °C with ZnCl_2_ as the catalyst. Calcined carbon and AC were identified via Raman spectroscopy. Subsequently, the composites were prepared using a physical mixing method. AC and its composites, containing PANI-AC1 and PANI-AC3, were characterized via FTIR spectroscopy, as well as BET, SEM, and CV. The H_2_S-sensing performance of PANI and its composites was investigated herein. Notably, the PANI-AC3 composite showed a higher sensing response to H_2_S with higher repeatability and stability than neat PANI. The increased H_2_S-sensing response of the PANI-AC composites was attributed to the large surface area of AC, which provided abundant active sites, resulting in an enhanced redox and doping capacity. The response of the sensors based on PANI-AC1 and PANI-AC3 increased by 1.34 and 3.16 times, respectively. Moreover, the as-prepared sensors exhibited a high sensing selectivity toward H_2_S, with minimal to negligible responses toward other gases. Therefore, the development of PANI-AC gas sensors holds great promise for the recycling and utilization of agricultural waste and for advancing the field of gas sensing.

## Figures and Tables

**Table 1:** BET analysis of CC, AC, PANI, PANI-AC1, and PANI-AC3.

Sample Code	S_BET_ (m^2^/g) ^a^	V_t_ (cm^3^/g) ^b^	D_p_ (nm) ^c^
CC	5	0.01	42.7
AC	1718	1.10	2.6
PANI	38	0.21	22.8
PANI-AC1	225	0.43	16.6
PANI-AC3	416	0.93	4.2

^a^ BET surface area. ^b^ Total pore volume calculated from P/P_0_ = 0.99. ^c^ Pore diameter.

**Table 2:** Conductivities of the undoped, doped, dedoped, and redoped forms of PANI, PANI-AC1, and PANI-AC3.

Sample Code	Conductivity (S/cm)			
	Undoped	Doped	Dedoped	Redoped
PANI	6.02 × 10^−6^	2.24 × 10^−3^	6.42 × 10^−6^	2.2 × 10^−3^
PANI-AC1	6.35 × 10^−6^	4.96 × 10^−3^	7.07 × 10^−6^	4.46 × 10^−3^
PANI-AC3	7.60 × 10^−6^	3.87 × 10^−2^	7.81 × 10^−6^	3.65 × 10^−2^

**Table 3:** Performance comparison of other conductive polymers-based H_2_S sensors with different morphology with the present work.

Sample Code	Response (R_a_/R_g_)	Gas Concentration (ppm)	Operating Temperature (°C)	Ref.
PANI-NF	7.32	25	150	[62]
PTh-WO_3_	1.4	200	70	[63]
PPy film	5	10	25	[64]
Ti_3_C_2_T_x_/PDS-Cl	2.2	5	30	[65]
PVA-IL-WO_3_	15	10	80	[66]
Ag NPs-PPy NTs	1.3	20	25	[67]
PPy microwire	1.85	200	25	[68]
PPy/WO_3_	1.61	1000	90	[69]
PANI-AC3	8.6	10	25	This work

## Data Availability

The data that support the findings of this study are available from the corresponding author, but restrictions apply to the availability of these data, which were used under license for the current study, and so are not publicly available. The data are, however, available from the authors upon reasonable request and with permission of the funding party, Ministry of Science and Technology, Taiwan, R.O.C.
